# Complete genome sequences of mycobacteriophages Ageofdapage, Aubs, BABullseye, CheetoDust, ShaboiShabazz, and TomBrady

**DOI:** 10.1128/mra.01253-23

**Published:** 2024-02-09

**Authors:** Shelbie Bass, Danielle Coates, Nina Frasketi, Smeetha James, Vishnu Marella, Riya Pulla, Kyle Stoecker, Srichandra Tupurani, Allison A. Johnson

**Affiliations:** 1 Center for Biological Data Science, Virginia Commonwealth University, Richmond, Virginia, USA; Portland State University, Portland, USA

**Keywords:** bacteriophage, *Mycobacterium*, mycobacteriophage

## Abstract

We report genome sequences of six mycobacteriophages. Each virus was isolated from a soil sample and belongs to the siphovirus morphology. Genomes are 41,901–60,613 bp in length, contain between 62 and 103 protein-coding genes, with up to 40% of those genes having a predicted function.

## ANNOUNCEMENT

The bacterial phylum Actinobacteria comprises a diverse group of gram-positive bacteria that are found in a variety of ecosystems (1). Some Actinobacteria cause illness in humans (1), and viruses, or bacteriophages, that infect this phylum have now been used in phage therapy treatments for at least 20 patients with *Mycobacterium* infections (2). We are interested in understanding the genomic diversity of phages infecting Actinobacteria so that phage therapy can be improved.

For more than 15 years, the SEA PHAGES program has engaged undergraduate students in Actinobacteriophage discovery and bioinformatics with the goal of improving STEM education while increasing knowledge of phage evolution and sequence diversity. Here, we report the genome sequences of six mycobacteriophages isolated from surface-level soil samples collected in Richmond, Williamsburg, and Aldie, Virginia (Table 1). Each bacteriophage was discovered through enrichment with the host bacteria *Mycobacterium smegmatis* mc2155 (NRRL 24020) (3). Enrichment samples were grown overnight at 37°C using 7H9 media. Phages were purified through two to six rounds of plaque picking, serial dilution, infection into cultures, and plating with top agar (3). Each phage produced small, round plaques of varying clarity. Negative staining using 1% uranyl acetate for transmission electron microscopy revealed siphovirus-like morphology.

**TABLE 1:** Phage and genome sequence characteristics

Phage	Aubs	ShaboiShabazz	BABullseye	TomBrady	CheetoDust	Ageofdapage
Sample location (city, state)	Aldie, VA	Richmond, VA	Williamsburg, VA	Richmond, VA	Richmond, VA	Richmond, VA
Sample location GPS coordinates	38.92169 N, 77.55269 W	37.5464 N, 77.4536 W	37.234482 N, 76.67864 W	37.548472 N, 77.45375 W	37.545141 N, 77.454741 W	37.545273 N, 77.454804 W
Plaque morphology	Clear	Clear	Halo with clear center	Cloudy	Cloudy	Clear
Plaque size	1 mm	1 mm	3.5–4 mm	1 mm	1 mm	3 mm
Capsid size (nm)	76 (*n* = 2)	55 (*n* = 2)	60 (*n* = 1)	57 (*n* = 2)	50 (*n* = 1)	50 (*n* = 2)
Tail length (nm)	205 (*n* = 2)	175 (*n* = 2)	136 (*n* = 1)	193 (*n* = 2)	205 (*n* = 1)	255 (*n* = 2)
SRA accession number	SRR27188678	SRX22868882	SRX22868879	SRX22868883	SRX22868880	SRR2718860
No. of reads	487,877	642,024	924,556	944,512	66,355	541705
Approximate read coverage	1,169	2,158	2,589	3,187	77	1,284
GenBank accession number	OP297542	ON637759	OP434447	ON970618	OR521075	OR521076
Cluster	F1	G1	A6	G1	K1	K1
Genome length (bp)	58,937	41,901	50,450	41,902	59,302	60,613
# ORFs (# with predicted function)	103 (40)	62 (25)	97 (35)	63 (25)	96 (39)	100 (38)
# Orphams	0	0	1	0	0	2
# tRNAs	0	0	3	0	1	1
% GC	61.4	66.6	61.5	66.6	66.5	66.5
Length of 3′-sticky overhang	10 base	11 base	10 base	11 base	11 base	11 base

DNA was isolated from phage high-titer lysate using the Promega Wizard DNA Cleanup Kit. Genomic DNA was sequencing using an Illumina MiSeq sequencer (v3 reagents) after preparing individual libraries using the NEBNext Ultra II FS Kit. The number and approximate coverage of single-end 150 bp reads are listed in Table 1. Raw reads were assembled into a single contig for each genome using Newbler v2.9 with default parameters. Genomes were visually checked for completeness using Consed v20 (4).

The resulting genomes were 41,901–60,613 bp in length, with a GC content of 61.4%–66.6% (Table 1). Assemblies yielded single contigs with ends containing 10–11 base 3′-sticky overhangs that were evident by many reads ending at the same position. Genomes were assigned to Actinobacteriophage clusters A6, F1, G1, and K1 according to established guidelines (5, 6).

Phage genes were predicted by Genemark v2.5 (7) and Glimmer v3.02 (8) using PECAAN (9) and DNA Master v5.23.6 (http://cobamide2.bio.pitt.edu). tRNA genes were predicted using Aragorn v1.2.38 (10) and tRNAscanSE v2.0 (11). Proteins containing membrane-spanning domains were identified using TMHMM (12) and TOPCONS (13). Potential protein functions were predicted using HHPred [using the PDB_mmCIF70, SCOPe70, Pfam-A, and NCBI_Concerved_Domains databases (14)] and Blastp [NCBI non-redundant database (15)]. After auto-annotation, students manually curated predicted positions and function for each gene (16).

Genomes contained between 62 and 103 predicted genes, with 36%–40% of those genes having a predicted function (Table 1). tRNA genes were identified in BABullseye (3), CheetoDust (1), and Ageofdapage (1). Three genes out of a combined 521 total predicted protein-coding genes in these genomes were determined to be orphams or novel genes with no sequence homologs within the Actinobacteriophage database (6, 17). All six phages are predicted to be temperate phages based on genome content and cluster designation.

## Data Availability

The annotated genome sequences for each phage are available at GenBank: Ageofdapage (OR521076), Aubs (OP297542), BABullseye (OP434447), CheetoDust (OR521075), ShaboiShabazz (ON637759), and TomBrady (ON970618). Sequencing reads are available at the Sequence Read Archive (SRA) through accession numbers SRX22868876, SRX22868878, SRX22868879, SRX22868880, SRX22868882, and SRX22868883.
